# Sulfonated polystyrene nanospheres from waste sources for the extraction of sulfonamide antibiotics from complex matrices

**DOI:** 10.1007/s00604-026-07875-1

**Published:** 2026-02-16

**Authors:** Lorenzo Antonelli, Ángela Inmaculada López-Lorente, Alessandra Gentili, Rafael Lucena, Soledad Cárdenas

**Affiliations:** 1 Affordable and Sustainable Sample Preparation (AS₂P) research group, Departamento de Química Analítica, Instituto Químico para la Energía y el Medioambiente IQUEMA, Universidad de Córdoba, Campus de Rabanales, Edificio Marie Curie, Córdoba, E-14071 Spain; 2 Department of Chemistry, Sapienza University, P.le Aldo Moro 5, Rome, 00185 Italy

**Keywords:** Plastic waste upcycling, Sulfonamides antibiotics, Polystyrene sulfonated microspheres, Zero waste extraction, In-syringe dispersive-SPE

## Abstract

A next-generation sorbent phase is introduced based on recycled polystyrene microspheres sulfonated with sulfuric acid and its application to the extraction of four representative sulfonamides (i.e., sulfanilamide, sulfaguanidine, sulfadiazine, and sulfamerazine) from biological matrices. The variables related to the sulfonation process and those affecting the extraction process have been evaluated. The in-syringe miniaturized dispersive solid-phase extraction method coupled to liquid chromatography-tandem mass spectrometry provided limits of detection of 0.8 µg L^− 1^ for sulfaguanidine in urine to 32 µg L^− 1^ for sulfanilamide in saliva. The precision, expressed as relative standard deviation has been evaluated at four concentration levels both intra-day and inter-day, being lower than 18.3 and 14.3% at the lower limit of detection, respectively. The accuracy, calculated with spiked urine and saliva samples and expressed as relative recovery, ranged from 86.4 to 108.5%. This strategy integrates sustainable waste recovery with the development of a high-performance, low-impact adsorbent, employing recycled materials (such as syringes and frits) to create a nearly zero-waste extraction system. This combination of recycling, analytical performance, and sustainability supports the evolution of environmental analysis toward a “white circular” model, seamlessly merging Green Science with robust analytical standards.

**Graphical abstract:**

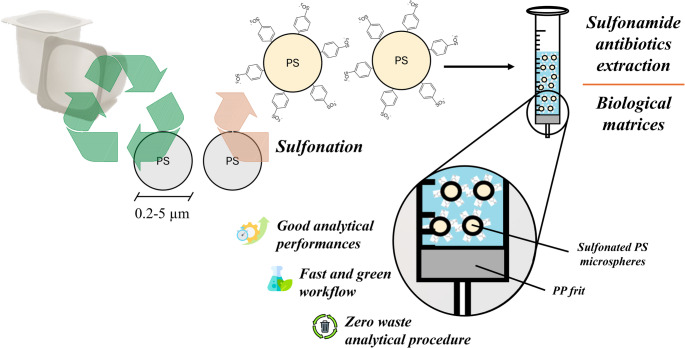

**Supplementary Information:**

The online version contains supplementary material available at 10.1007/s00604-026-07875-1.

## Introduction

 Sulfonamide antibiotics are a class of synthetic antimicrobial agents characterized by the presence of a sulfonamide functional group. These compounds act as competitive inhibitors of dihydropteroate synthase, an enzyme responsible for a key step in folic acid biosynthesis [1]. Folic acid is an essential precursor for DNA and RNA synthesis and plays a crucial role in several biochemical pathways associated with cellular proliferation [2]. By disrupting folic acid production, sulfonamides exert a bacteriostatic effect against a broad spectrum of Gram-positive and Gram-negative bacteria.

According to the 2022 ESVAC report published by the European Medicines Agency, sulfonamides rank as the third most widely traded class of veterinary medicinal products [3]. However, their extensive application has raised significant concerns due to their persistence, bioaccumulation potential, and capacity to act as endocrine disruptors [4]. Given the environmental and health risks associated with sulfonamide contamination, the development of innovative analytical methods and next-generation materials capable of extracting these compounds from complex matrices is of paramount importance. In particular, sustainable and cost-effective analytical approaches are increasingly sought after.

From a biological perspective, sulfonamides are primarily excreted in urine and can also be present in saliva, mostly in their unchanged form [5]. The inclusion of both matrices allows the method to be evaluated in different relevant biological fluids and demonstrates its versatility. From a chemical perspective, sulfonamide antibiotics contain an aromatic amino group and a sulfonamide moiety, and in biological fluids they mainly exist in pH-dependent acid-base equilibria. They behave as weak acids associated with the sulfonamide proton, while the aromatic amino group is weakly basic due to electron delocalization within the benzene ring and therefore remains largely non-protonated under physiological conditions (see Table S1 of the Supplementary material) [6, 7].

Solid-phase extraction (SPE) remains a well-established and widely adopted technique for the preconcentration of sulfonamide antibiotics. Its effectiveness has been demonstrated, for instance, by the on-site extraction of seven target sulfonamides using a 3D-printed device functionalized with Oasis MCX resin, followed by high performance liquid chromatography diode array detection analysis [8]. In addition, Oasis HLB cartridges have been extensively reported as efficient extraction media for the isolation of sulfonamide antibiotics prior to liquid chromatography tandem mass spectrometry determination, enabling their detection in wastewater samples at concentration levels ranging from 0.03 to 0.07 µg L^− 1^ [9]. To address matrix interferences associated with complex environmental samples, combined SPE strategies have also been proposed. In particular, a tandem configuration of strong anion-exchange (SAX) and HLB cartridges has been employed for soil extract clean-up, allowing the selective removal of negatively charged humic substances by the SAX phase while retaining antibacterial agents on the HLB sorbent [10]. These approaches highlight the importance of properly selecting the sorbent nature to achieve optimal analyte isolation and detection.

Dispersive techniques, such as liquid-liquid-liquid microextraction (LLLME), have also been applied for sulfonamide determination in water samples before and after wastewater treatment, achieving quantification limits as low as 0.22 µg L^− 1^ and highlighting the partial removal of these compounds during treatment processes [11].

Building on these traditional methods, several innovations have recently emerged, focusing on miniaturization, portability, and enhanced environmental sustainability. Notably, graphene nanoplatelet-packed pipette-tip micro-solid phase extraction (PT-µSPE) has been integrated with smartphone-based fluorescence detection for rapid screening of four sulfonamides, offering a low-cost, eco-friendly analytical platform with recoveries between 94% and 102% and detection limits below 3.1 µg L^− 1^ [12]. Electrochemical sensing has also gained attention: nitrogen-doped Cu-based metal–organic frameworks have shown excellent surface properties (∼1184 m^2^ g^− 1^) and detection limits as low as 0.003 µM for sulfanilamide, supporting their application in fast, highly sensitive pollutant monitoring [13]. Polymeric adsorbents continue to offer competitive extraction performance, with magnetic polystyrene sulfonate sodium material developed for the selective removal of sulfonamides such as sulfamerazine and sulfafurazole, demonstrating high adsorption capacity and ease of magnetic recovery [14].

In this context, the present study explores, for the first time, the use of recycled waste polystyrene (PS) as a cost-effective and sustainable sorbent for the extraction of sulfonamide antibiotics from biological matrices. Waste-derived PS was solubilized in an organic solvent and subjected to an emulsion solidification process, yielding uniformly shaped microspheres [15], which were then sulfonated using concentrated sulfuric acid. Several sulfonation strategies for polystyrene have been reported in the literature, aiming to introduce sulfonate functionalities for enhanced adsorption and extraction capabilities. Traditional sulfonation protocols often employ concentrated sulfuric acid [16], but also acetyl sulfate [17, 18], sulfur trioxide [19] and silica sulfuric acid [20] have been reported as sulfonating agents. Due to the versatility of sulfonated PS derivatives in adsorption and analytical extraction, this material has been integrated into advanced microextraction platforms. For example, magnetic Fe_3_O_4_@PS microspheres prepared via suspension polymerization have shown efficient magnetic solid-phase extraction (MSPE) performance for food safety contaminants [21]. Composite materials incorporating sulfonated PS with carbon nanotubes have also been designed for PT-µSPE with effervescence-driven dispersion, targeting alkaloids and flavonoids in herbal matrices [22]. These examples support further exploration using recycled polystyrene waste; the present work presents the first synthetic approach that simultaneously achieves effective waste PS recycling and the development of a high-performance sorbent for sulfonamide extraction from real-world matrices.

A key innovation of this methodology is its commitment to a fully circular [23], waste-minimizing approach. The entire extraction system, ranging from the adsorbent material to the support components, relies exclusively on recycled materials. The polystyrene source, the syringe used for extraction, and the frit serving as the physical filter for the sulfonated PS microspheres are all derived from repurposed waste. This strategy represents one of the first extraction techniques to propose an integrated recycling model, achieving near-zero waste generation while maintaining high analytical performance.

## Experimental section

### Materials and reagents

All reagents were of analytical grade or better. Sulfadiazine, sulfamerazine, sulfanilamide, sulfaguanidine, sodium chloride and sodium dodecyl sulfate (SDS) were supplied by Sigma-Aldrich (Madrid, Spain, www.sigmaaldrich.com). Stock standard solutions were prepared in methanol (MeOH, Panreac, Barcelona, Spain, www.itwreagents.com/iberia/es/home) at a concentration of 1 mg mL^− 1^ and stored at 4 °C in the dark. Working solutions were prepared by dilution of the stock in MeOH or Milli-Q water (Millipore Corp., Madrid, Spain, www.sigmaaldrich.com), as required. Both the optimization study and the validation workflow have been performed with microspheres synthetized departing from waste polystyrene, coming from different commercial yogurt cups, pooled. Ethyl acetate, ethanol, sulfuric acid (95–98%) ammonia (30%) and formic acid (≥ 98%) were purchased from Panreac.

### Real samples

Saliva samples were collected by passive drooling from ten healthy volunteers, aged between 23 and 27 years, within our laboratory. None of the participants were taking medications containing sulfonamides at the time of sample collection. The samples were used as blank matrices and subsequently spiked with the target analytes for method validation. All the collected blank samples have been pooled together and stored at 4 °C until analysis (within 3 days from the collection). Aliquots of the pooled saliva sample have been centrifuged, after pH regulation to 3.5 with formic acid (to ensure the entire elimination of precipitating proteins). The supernatant was withdrawn, eliminating the solid residue. The sample was diluted 1:1 (v/v) with MilliQ water to ensure a reproducible and complete dispersion of the sulfonated polystyrene (sPS) microspheres in the extraction media. The pH was checked once again to ensure that the extraction will be conducted at pH 3.5.

Similarly, eight blank urine samples were obtained from the same volunteers. They were pooled, subsampled (50 mL fractions) and stored in fridge (used within 3 days form the collection). The samples were 1:1 (v/v) diluted in MilliQ water and the pH adjusted at 3.5 with formic acid. No real samples from individuals treated with these drugs were available for this study.

### Preparation of PS microbeads and sulfonation procedure

This synthesis involves scaling up the procedure previously proposed by our group in an earlier publication [24]. A 2.5% (w/v) solution of grinded PS (sourced from pooled waste yogurt containers) in ethyl acetate was prepared, with dissolution facilitated by gentle magnetic stirring.

A 10 mL portion of this solution was transferred into a 200 mL glass bottle containing a magnetic stir bar. For the emulsion-solidification process, two aqueous solutions were separately prepared: one saturated with NaCl and another containing 1% (w/v) sodium dodecyl sulfate. These solutions were sequentially added to the glass bottle (20 mL of the NaCl solution followed by 50 mL of the SDS solution), forming a biphasic system, which was maintained as a microemulsion by magnetic stirring at 900 rpm.

To destabilize the emulsion and precipitate PS in the form of microbeads, 50 mL of absolute ethanol was rapidly added while the system was stirring. The resulting PS microbeads were collected by filtration using a circular glass fiber filter mounted on a vacuum pump, allowing the supernatant to gently flow through while retaining the dispersed microspheres on the filter surface. The microparticles were then sequentially washed with water and ethanol until the washing solvent appeared clear and free of foam. Finally, the PS microspheres were dried overnight at 45 °C.

**Fig. 1:**
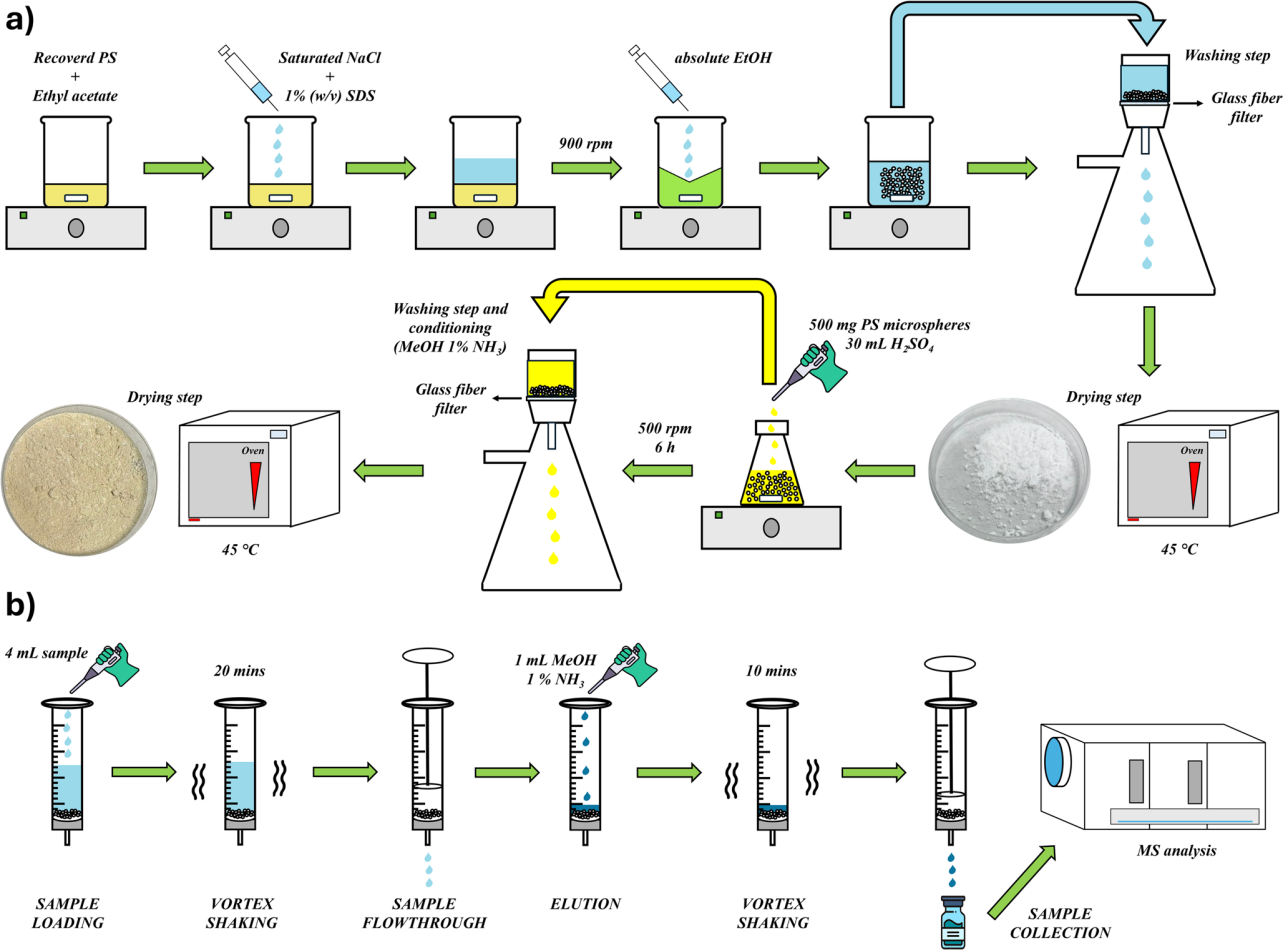
Schematic representation of the synthetic route of the PS-P (**a**) and the analytical procedure for the extraction and MS/MS determination of the sulfonamides (**b**)

Once dried, the material underwent a sulfonation process. Specifically, 500 mg of PS microspheres were weighed directly into a 50 mL conical flask, followed by the addition of 30 mL of concentrated sulfuric acid (95–98%). The reaction was allowed to proceed under gentle magnetic stirring for a duration ranging from 1 to 16 h, an interval optimized to balance performance and process time. This reaction, already documented in the literature [25], is illustrated in Fig. S1. Following sulfonation, the sPS microspheres were filtered using a glass fiber filter mounted on a vacuum pump to remove the reaction medium. The material was then sequentially washed with 10 mL of Milli-Q water, 10 mL of ethanol, and 10 mL of MeOH containing 1% ammonia (same composition of the eluent solution) to eliminate reaction by-products and equilibrate the sPS microspheres in its deprotonated form. Finally, the material was dried overnight in an oven at 45 °C, rendering it ready for application. The whole synthesis yield of the final material is almost 100% (in terms of final weight of microbeads with respect to the weight of employed PS). Figure 1a displays a scheme of the whole procedure.

### Characterization of the sPS microspheres

sPS microbeads were studied after the synthesis by attenuated total reflection Fourier transformed infrared spectroscopy (ATR-FTIR), scanning electron microscopy (SEM) and energy-dispersive X-ray spectroscopy (EDX).

Microbeads, both before and after sulfonation process, were characterized in terms of dimensional and morphological properties via SEM using a JEOL JSM 7800 F microscope (Akishima, Tokyo, Japan, www.jeol.com) available at the Central Service for Research Support of the University of Córdoba. Prior to scanning, all samples were coated with gold to make their surfaces conductive. Micrographs were collected at a magnification of 10000x. With the same instrumentation, via a specific detector for EDX, the material was analyzed in terms of chemical composition and elemental distribution.

ATR-FTIR spectra were collected by using a Thermo Fisher Nicolet Apex FT-IR (Thermo Fisher, Milano, Italy, www.thermofisher.com), equipped with a synthetic diamond ATR cell. Spectra were collected with a spectral window of 4000–550 cm^− 1^, coadding 150 scans for each sample. Data collection and processing were made using the OMNIC software package (Thermo Fisher, Milano, Italy). By the comparison of the spectroscopic profiles of the material before and after the sulfonation process, it was possible to assess the efficiency of the chemical modification of the polymeric substrate.

### Analytical procedure

The extraction procedure was developed and optimized inspiring to an in-syringe miniaturized dispersive solid-phase extraction IS-dSPE. 20 mg of the sPS microspheres were transferred to a syringe system, where all the extraction process is driven. The syringe is equipped with a polypropylene frit, used as a physical filter for sPS. Both the syringe and the frit are reusable, allowing a 100% recyclable extraction process, from the adsorption material to the support devices. For the extraction, 4 mL of sample (i.e., saliva, urine, both 1:1 diluted with MilliQ water) containing the analytes at concentrations within the linear range were transferred to the syringe system. The sPS was allowed to disperse inside the sample for 20 min, maximizing the mass transfer to the sorbent, by the vortex agitation. Next, pressure was applied with a syringe plunger to allow the sample to flow through the frit. After the sample loading, sPS microbeads were washed with 1 mL of MilliQ water to ensure the elimination of matrix interferences not physically retained on the sorbent. Analytes were then eluted by adding 1 mL of MeOH 1% in ammonia to the body of the syringe, assisting the process with 10 min of vortex agitation. A manual pressure was finally applied, and the eluent was collected in a vial, ready for subsequent instrumental analysis. The entire analytical procedure is displayed in Fig. 1b.

Analyses were conducted using high performance liquid chromatography separation coupled with a mass spectrometry MS/MS triple quadrupole system (Agilent 1260 Infinity HPLC system, Agilent, Palo Alto, CA, USA, www.agilent.com). The system was equipped with a binary high-pressure pump for mobile phase delivery and an autosampler. C18 stationary phase was selected, in the form of an Eclipse Plus C18 (3.5 μm, 4.6 × 100 mm, from Agilent, Palo Alto, CA, USA). Chromatographic separation was performed in isocratic mode, using a mobile phase consisting of 90% H₂O with 0.1% (v/v) ammonia and 10% MeOH. The mobile phase flow rate was maintained at 0.3 mL min^− 1^, with a total analysis time of 6.5 min.

Target analytes were quantified in multiple reaction monitoring (MRM) mode using an Agilent 6420 Triple Quadrupole MS (Agilent, Palo Alto, CA, USA) with an electrospray ionization (ESI) source. The optimized ion transitions, chromatographic retention times and physicochemical descriptors for each analyte are listed in Table S1. The drying gas (N₂, 99% purity) was set at a flow rate of 10 L min^− 1^ and a temperature of 300 °C. The nebulizer pressure was 18 psi, and the capillary voltage was maintained at 2000 V in positive ionization mode for all analytes. Data acquisition and processing were performed using Agilent MassHunter Software (Version B.06.00).

## Results and discussion

### Optimization of synthetic parameters

The procedure for preparing PS-based microbeads from recycled material was adapted from a previously published methodology [24]. A scale-up of the synthesis was undertaken to enhance productivity while preserving the reproducibility and morphological consistency of the resulting microbeads. The outcomes of the scaled-up process yielded a material morphologically comparable to the original.

Sulfonation of the PS substrate was carried out via batch reaction with concentrated sulfuric acid, a widely established method in the literature [16, 25–28]. In this study, the protocol was applied to a polymeric substrate derived from waste materials, with the aim of improving its affinity for the target analytes.

A critical factor in the sulfonation process is the contact time between the PS microparticles and sulfuric acid. The impact of sulfonation was assessed by evaluating the retention capacity of the material as a function of reaction time. Adsorption experiments were conducted by putting in contact the adsorption phase with a synthetic water sample spiked with four sulfonamide compounds at a concentration of 100 µg L^− 1^. For these preliminary tests, the pH was adjusted to 3 by adding formic acid to Milli-Q water. At this pH value, sulfonamide antibiotics are either in their protonated form or neutral. The sorption was performed in-syringe, following the initial steps of the extraction protocol. After contact with the sorbent phase, the water sample was directly analyzed to quantify the fraction of analytes not retained by the material.

**Fig. 2:**
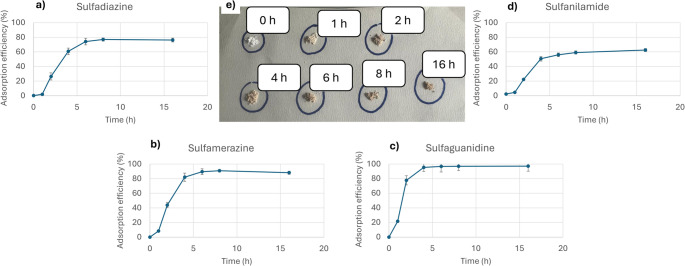
Effect of sulfonation time on the adsorption efficiency (%) for each analyte: (**a**) sulfadiazine, (**b)** sulfamerazine, (**c)** sulfaguanidine and (**d)** sulfanilamide. The image inset (**e**) illustrates the visual change in microsphere color resulting from sulfuric acid treatment

As shown in Fig. 2, all analytes exhibited a consistent trend: the concentration of unretained compounds decreased with increasing sulfonation time. Beyond six hours of sulfonation, no statistically significant improvement in retention was observed, indicating that the material had reached a saturation point, likely corresponding to its maximum surface sulfonation. These findings demonstrate that the raw material exhibited negligible affinity for the selected sulfonamides, while sulfonation markedly enhanced adsorption performance. A reaction time of six hours was therefore selected as the optimal condition to achieve full sulfonation and maximize the material’s retention capacity.

### Characterization of the sPS

**Fig. 3:**
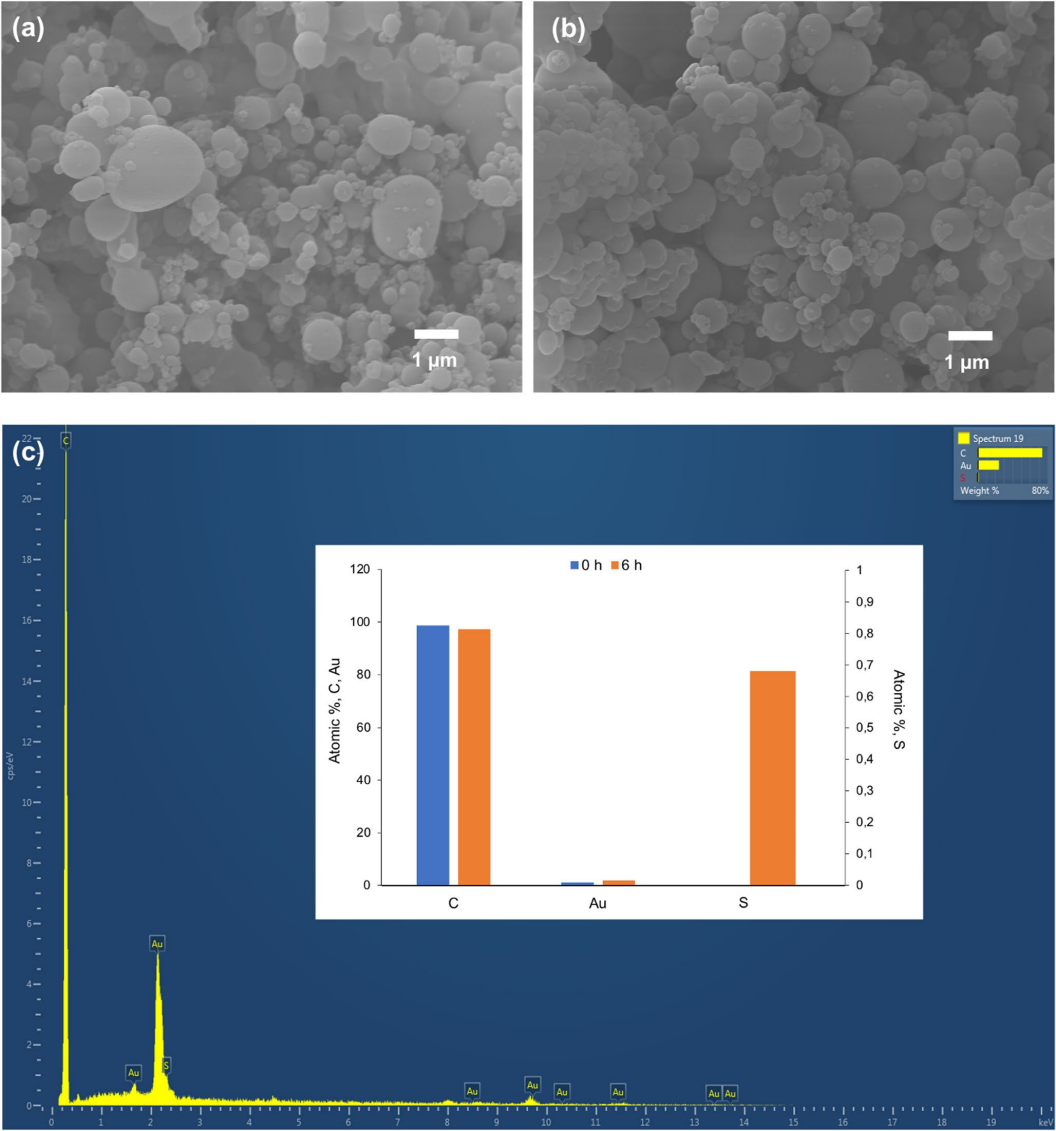
SEM images of PS microbeads obtained through the emulsion solidification process (**a**) raw PS microbeads without sulfonation, (**b**) PS microbeads treated with sulfuric acid for 6 h. Micrographs were acquired with a magnification of 10000x. (**c**) EDX analysis of the materials. The inset depicts the atomic percentage of the materials (samples were covered with Au prior SEM-EDX analysis to improve sample conductivity). Left axis corresponds to atomic % of C and Au and right axis that of S

SEM analysis was conducted to verify the shape and size uniformity of the synthesized material, while EDX analysis was also performed to analyze the elemental composition. For comparison, both the non-sulfonated material and the material subjected to a 6-hour sulfonation process were analyzed. The microbeads obtained with the scaled-up synthesis are, similarly to those previously described, polydisperse in size, observing fine particles with a diameter ranging from 0.1 to 1 μm as well as coarse particles with diameters > 1 μm (Fig. 3a). The sulfonation procedure modified neither the shape nor size distribution of the PS microbeads (Fig. 3b), while EDX analysis evidenced the presence of S within the surface of the material, although with a low atomic %, the material being composed primarily by C (Fig. 3c). It should be noted that the presence of Au within the EDX spectra arises from the gold layer deposited prior SEM analysis to improve the conductivity of the sample and, thus, the acquisition of SEM images.

ATR-FTIR analysis was carried out to rule out degradation of the bulk polymer and to confirm the surface sulfonation induced by sulfuric acid. For comparison, three materials were analyzed: polystyrene waste, pristine polystyrene microbeads prior to sulfonation, and the final sPS microbeads. Their spectra are presented in Fig. S2, with band assignments and detailed descriptions available in the Supporting Information. The spectrum of the final material shows complete preservation of the polymeric backbone, with only minimal changes in the spectroscopic profile before and after emulsion solidification and sulfonation. Neither solvent dissolution nor acid treatment appears to alter the chemical composition of the polymeric support. As a second key observation, typical bands corresponding to sulfonate groups are not clearly detectable (expected at ~ 1030–1080 cm^− 1^ and ~ 1120–1170 cm^− 1^ for symmetric and asymmetric S = O stretching vibrations, and ~ 1350 cm^− 1^ for additional S = O modes). This can be explained by the superficial nature of the functionalization and the preserved predominance of the bulk polymer’s characteristic functional groups, which tend to overshadow the spectroscopic evidence of the newly introduced sulfonate groups.

### Optimization of extraction methodology

The variables influencing the extraction procedure were subjected to an optimization study, including the sample solution volume, the amount of sPS in the syringe, pH, ionic strength, and the composition and volume of the desorption phase. All optimization experiments were performed using spiked Milli-Q water samples, with each analyte at a concentration of 100 µg L^− 1^.

Before specific optimizations, the general conditions were as follows: pH = 3, ionic strength = 0.15 µS/cm (no sodium chloride added), eluent phase = MeOH 0.1% aqueous ammonia, and eluent volume = 1 mL.

Initially, extraction trials were conducted without adding sPS in the syringe to evaluate the background extraction efficiency of the device. The results of this preliminary test, reported in Fig. S3 of the Supplementary Material, indicate that analyte retention on the syringe walls and within the polypropylene frit porosity was negligible, resulting in an extraction yield below 2% of the LLOQ extract.

**Fig. 4:**
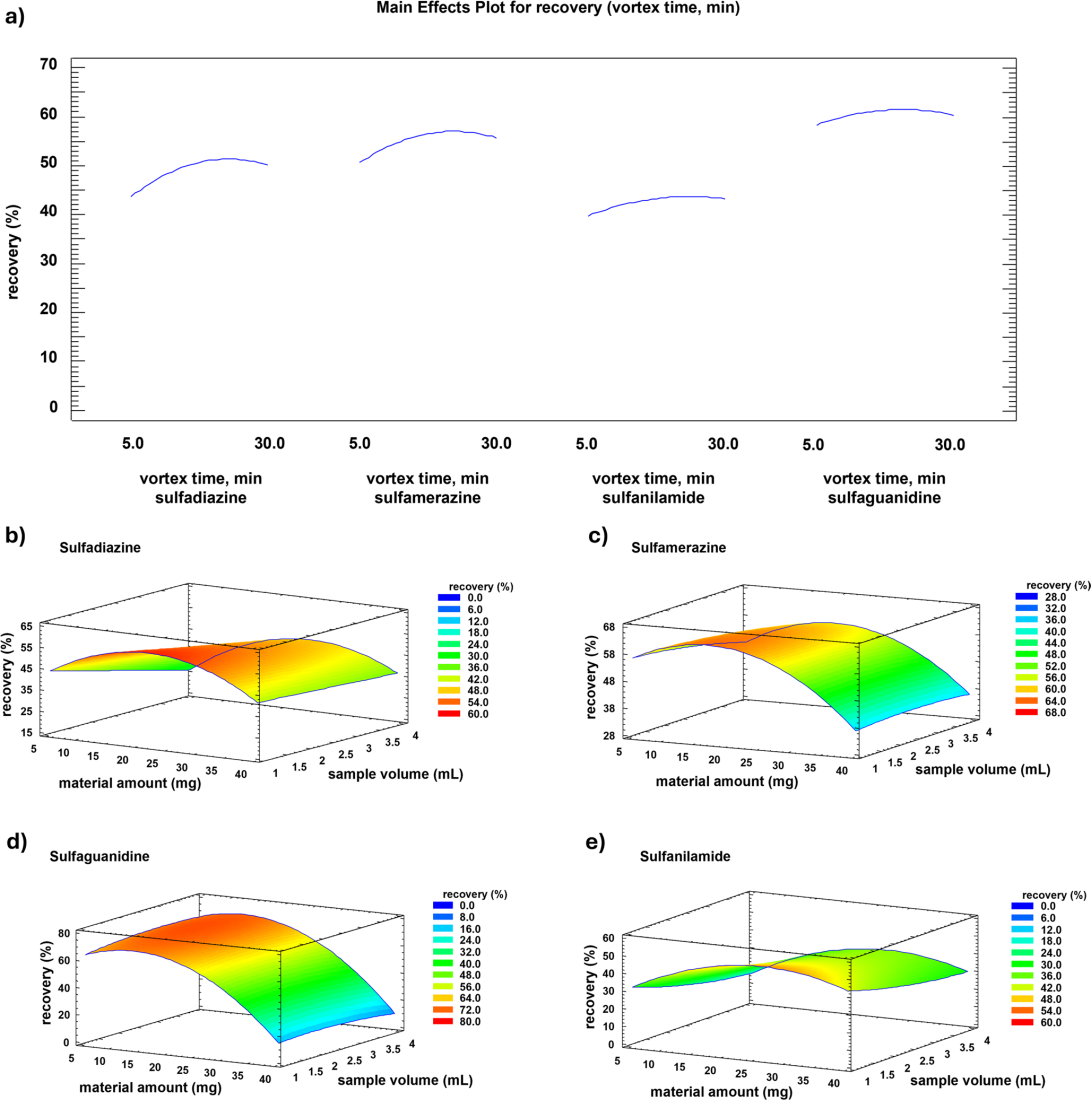
Main effects plot (**a**) for vortex time and response surface plots showing the influence of sorbent amount and sample volume on extraction recoveries for each analyte. For the response surface analyses (**b** for sulfadiazine, **c** for sulfamerazine, **d** for sulfaguanidine and **e** for sulfanilamide), vortex time was fixed at the optimized value of 20 min

Response Surface Methodology (RSM) was applied to assess the relationship between variables influencing the adsorption step of the extraction procedure and their impact on extraction yields, i.e. sorbent amount, sample volume and vortex time. A 3^3^ Box–Behnken design was employed for the optimization. Among all parameters, vortex time was identified as a critical factor affecting extraction performance. As shown in the main effect plot (Fig. 4), adsorption reaches completion within 20 min, achieving the maximum extraction yield. Therefore, for the 3D response surface analysis, which evaluates the relationship between sample volume, material amount, and process desirability (in terms of recovery percentage), vortex time was fixed at 20 min. The response surface plot in Fig. 4 demonstrates a strong correlation between average recovery and material amount, with optimal extraction yields observed between 20 and 30 mg of sPS, depending on the specific sulfonamide. To ensure consistent performance across all analytes, a compromise amount of 20 mg was selected. In contrast, sample volume had a minimal effect on extraction yield, as it did not influence process desirability but played a role in determining the enrichment factor. Consequently, the highest tested sample volume was chosen as the optimal condition for the final extraction workflow and maximization of the method sensitivity. In summary, optimal adsorption parameters were set as follows: vortex time = 20 min, material amount = 20 mg, sample volume = 4 mL.

**Fig. 5:**
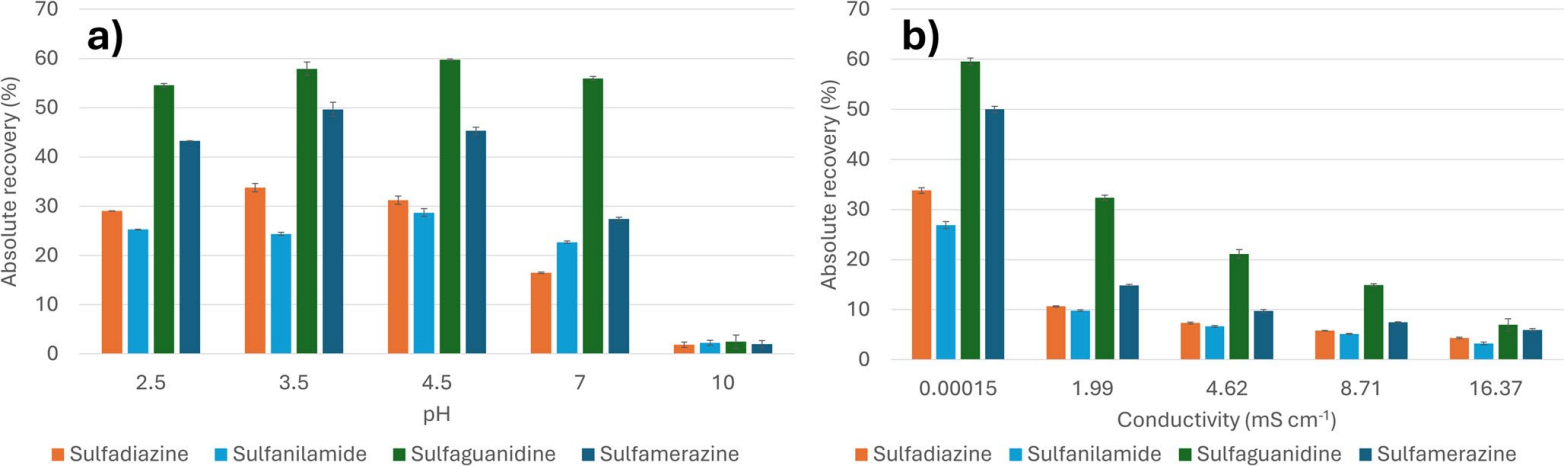
Effect of (**a**) pH and (**b**) ionic strength (expressed as conductivity) on the recovery (%) of target analytes. Experiments were performed using Milli-Q water spiked at 100 µg L^-1^ for each analyte. For the assessment of ionic strength, the pH was fixed at 3.5

Once the conditions for the adsorption step were established, the extraction procedure was further optimized by investigating the influence of pH and ionic strength on extraction yields from aqueous standards. The pH was adjusted using formic acid or ammonia and measured with a Horiba F-51 pH meter (Kyoto, Japan). Since the pH determines the ionization state of the analytes, it directly influences their interaction with the sorbent material. Extraction performance was evaluated at five different pH values (i.e., 2.5, 3.5, 4.5, 7.0, and 10.0), with each condition tested in triplicate. As shown in Fig. 5a, the extraction efficiency was highest at pH values ≤ 6, where all analytes exist predominantly in their neutral or protonated form. A pH of 3.5 was selected as optimal, yielding the highest average absolute recoveries.

The effect of ionic strength was assessed by analyzing spiked aqueous samples (100 µg L^− 1^) at pH 3.5 but with varying conductivity levels. Different amounts of NaCl have been added to different spiked water samples (i.e., 0, 0.1, 0.25, 0.5 and 1%), obtaining solutions with different measured conductance (0.00015, 1.99, 4.62, 8.71, 16.37 mS cm^− 1^, respectively). Each condition was tested in triplicate. As shown in Fig. 5b, extraction efficiency decreased with increasing conductivity, suggesting that competing cations interfere with analyte binding. Therefore, no salts were added to adjust the ionic strength before extraction, to maximize interactions between analytes and the active sites of the sorbent.

**Fig. 6:**
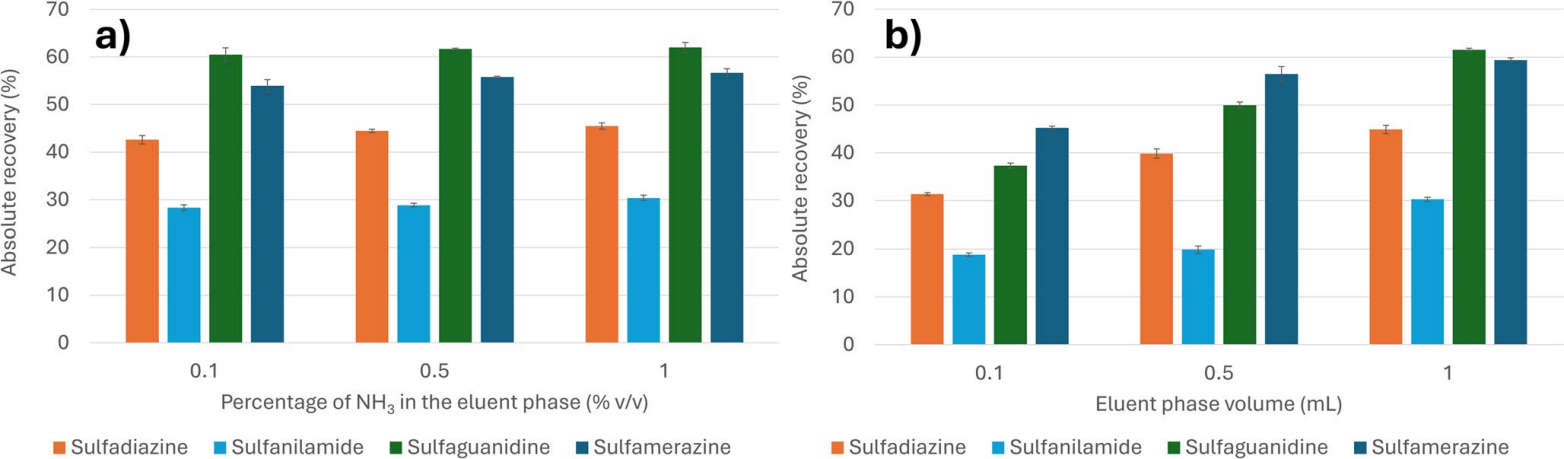
Results for the optimization of (**a**) the composition and (**b**) volume of the eluent phase (MeOH 1% NH_3_). Experiments were conducted on MilliQ water spiked at a fixed concentration of 100 µg L^− 1^ for each analyte and performing the entire adsorption step at the previous optimized conditions

The final set of parameters investigated concerned the elution conditions, specifically the nature and volume of the elution solvent. Preliminary experiments indicated that pure MeOH, either alone or supplemented with varying concentrations of aqueous ammonia, provided the best chromatographic separation. The inclusion of water in the elution mixture was found to reduce peak resolution. The addition of ammonia promotes a shift in the protonation equilibrium toward the neutral or even anionic form, facilitating desorption of the analytes from the resin. Consequently, three eluent compositions were evaluated: MeOH containing 0.1%, 0.5%, and 1% aqueous ammonia. As shown in Fig. 6a, only slight differences in recovery were observed among the tested conditions, with MeOH containing 1% aqueous ammonia yielding the highest overall recovery.

The influence of eluent volume on extraction efficiency was assessed in the range of 0.1–1 mL (Fig. 6b). Higher elution volumes improved analyte recovery by enhancing contact between the eluent and the sorbent, thus promoting more efficient desorption. An increase in precision was also noted with higher volumes. Volumes beyond 1 mL were not tested to avoid excessive dilution of the extract. Based on these findings, a volume of 1 mL MeOH containing 1% aqueous ammonia was selected for the subsequent validation protocol.

### Analytical features of the method

The analytical performance of the method was assessed through validation experiments, designed following ICH guideline M10 for bioanalytical method [29]. Table 1 presents the key figures of merit. Matrix-matched calibration curves were established for all analytes in two real biological matrices, i.e., pooled saliva and urine, using triplicate measurements. Blank samples were spiked at six to eight concentration levels (depending on the analyte-specific quantification limits) prior to extraction and subjected to the complete analytical workflow. Calibration curves were generated by plotting the mean peak area from triplicate analyses against the corresponding analyte concentrations (see Fig. S4). All analytes exhibited excellent linearity, with determination coefficients (R²) exceeding 0.99, across a dynamic range from the lower limit of quantification (LLOQ) up to 800 µg L^− 1^. This wide linear range ensures reliable quantification over a broad concentration spectrum, supporting the method’s applicability in diverse clinical and biological sample contexts. Figure S4 displays the calibration models for both saliva and urine.

**Table 1:** Figures of merit of the 4 sulfonamide antibiotics analyzed in spiked samples of urine and saliva. Precision and accuracy are reported at all tested concentration levels

Analyte	LOD (µg L^− 1^)	LLOQ (µg L^− 1^)	*R* ^2^	Linear range (µg L^− 1^)	RSD intra-day, *n* = 5 (%)				Accuracy, intra-day (% relative recovery, *n* = 5)	
					LLOQ	L-QC	M-QC	H-QC	LLOQ	L-QC
Urine										
Sulfadiazine	1.4	4	0.998	LLOQ-800	13.9	6.0	2.5	4.0	95.8	89.0
Sulfamerazine	1.9	4	0.996		11.4	5.6	4.9	4.2	102.3	91.7
Sulfaguanidine	0.8	2	0.998		18.3	4.9	5.8	4.0	105.8	108.5
Sulfanilamide	30	50	0.999		11.3	9.6	8.0	10.3	97.4	101.7
Saliva										
Sulfadiazine	3	5	0.991	LLOQ- 800	15.6	6.3	8.0	4.4	117.9	102.5
Sulfamerazine	4	5	0.991		10.1	9.0	8.0	3.1	115.8	101.6
Sulfaguanidine	1.3	4.8	0.996		9.2	6.9	10.5	2.9	86.4	101.7
Sulfanilamide	32	50	0.994		7.8	5.6	8.1	7.8	105.0	95.9

Analyte	Accuracy, intra-day(% relative recovery, *n* = 5)		RSD inter-day, *n* = 15 (%)				Accuracy, inter-day (% relative recovery, *n* = 15)			
	M-QC	H-QC	LLOQ	L-QC	M-QC	H-QC	LLOQ	L-QC	M-QC	H-QC
Urine										
Sulfadiazine	98.7	100.7	11.1	11.1	3.4	3.2	99.9	100.0	101.0	100.1
Sulfamerazine	100.5	98.7	8.8	12.1	4.5	3.6	101.9	105.8	100.9	95.7
Sulfaguanidine	108.5	107.0	14.3	7.0	6.0	4.3	100.8	99.7	100.7	104.5
Sulfanilamide	94.8	96.4	9.8	8.6	6.6	10.2	103.2	101.8	99.0	101.0
Saliva										
Sulfadiazine	102.6	102.4	10.3	3.8	3.5	4.8	95.7	104.2	105.9	98.7
Sulfamerazine	99.4	101.0	5.6	3.7	2.3	3.4	105.9	101.0	103.5	98.8
Sulfaguanidine	111.8	98.2	6.6	5.3	4.9	3.1	97.0	104.6	101.0	99.9
Sulfanilamide	106.3	104.1	8.4	4.3	4.2	8.3	102.4	100.0	99.2	96.8

Limit of detection (LOD) was determined as the concentration yielding a signal-to-noise ratio of 3 (S/*N* = 3), while the LLOQ was defined as the lowest analyte concentration ensuring precision and accuracy within 20%. As can be observed in Table 1, LODs were in the range 0.8 and 32 µg L^− 1^ for urine and saliva samples, while LLOQs ranged from 2 to 50 µg L^− 1^.

Precision and accuracy were assessed in accordance with ICH guidelines. For these studies a different pool of saliva and urine samples were employed, in which no presence of the analytes was found, so they were spiked at four fortification levels. The selected levels included: LLOQ, a low-quality control (L-QC) level (i.e., 2–3 times the LLOQ), a medium QC level (M-QC, approximately 30–50% of the calibration range), and a high QC level (H-QC, at least 75% of the upper calibration range). Precision was expressed as the relative standard deviation (RSD, %), while accuracy was evaluated through relative recovery. Both intra-run (within-run) and inter-run (between-run) precision and accuracy were evaluated. For intra-run analysis, five replicates were analyzed within a single analytical batch. For inter-run assessment, three independent analytical batches, each consisting of five replicates, were performed on three separate days. As summarized in Table 1, all RSD values were below 15% for all the analytes in the QCs levels and lower than 20% in the LLOQ concentration, meeting the acceptance criteria established by ICH guidelines. Relative recovery values ranged from 86.4% to 117.9%, demonstrating acceptable accuracy across all tested concentration levels.

### Comparison with other analytical methods

The most significant innovation introduced by this study lies in the full recyclability of all consumables used throughout the analytical workflow, combined with the implementation of an extraction sorbent derived entirely from recycled polystyrene. The approach yielded satisfactory analytical performance while drastically minimizing material waste. The use of recycled materials as sorbents in analytical chemistry is gaining increasing attention, with several studies confirming comparable performance to conventional synthetic sorbents. This work represents one of the few examples where these concepts have been applied in full, effectively aligning with the principles of “circular analytical chemistry” as theorized by Psillakis et al. [23], and translating them into a genuinely zero-waste and fully recyclable protocol. To confirm the complete reusability of all consumables, including the sorbent and plasticware, a series of reuse tests were carried out, as shown in Fig. S3 (Supplementary Material). Procedural blanks after washing routines using both water and acetone, or water alone, were analyzed. For all four sulfonamide antibiotics investigated, residual signals were below 3% of the signal obtained at the LLOQ, confirming that re-use does not compromise analytical integrity.

A comparison with previously reported methods for sulfonamide determination in complex matrices is presented in Table S2. Notably, while other approaches (e.g., QuEChERS, SPE, Molecularly Imprinted Polymer Solid-Phase Extraction (MISPE), and LLLME) often rely on single-use materials and generate significant waste, the current IS-DµSPE method uniquely combines low detection limits (i.e., 4–5 µg L^− 1^ for sulfadiazine and sulfamerazine, 50 µg L^− 1^ for sulfanilamide, and 2–4.8 µg L^− 1^ for sulfaguanidine) with short extraction time (~ 35 min) and full reusability of materials. This positions the method as a promising and sustainable alternative for sulfonamide quantification in biological matrices, bridging the gap between green chemistry and analytical reliability.

## Conclusions

This study underscores the transformative potential of integrating sustainability into the core of analytical method development. By designing a fully recycled extraction system (from the sorbent material to the structural components such as syringes and filters) this work introduces a truly circular, near-zero-waste analytical approach. The successful reuse and functionalization of waste-derived polystyrene, through sulfonation, demonstrates that post-consumer plastic materials can be upgraded into efficient and selective sorbents for the extraction of priority environmental contaminants such as sulfonamide antibiotics. Crucially, this strategy does not compromise analytical feasibility or performance. On the contrary, the method shows excellent efficiency and reproducibility in extracting the four studied sulfonamides from biological matrices. This confirms that sustainability and analytical robustness can go hand in hand, even when relying exclusively on recycled inputs. Furthermore, the functionalization of recycled materials adds a valuable dimension to waste management, shifting the paradigm from simple material reuse to the creation of tailor-made tools for specific analytical purposes. This aligns not only with green chemistry principles but also with broader goals of economic viability and social progress, offering low-cost, accessible solutions that can be adapted to various contexts, including resource-limited settings. Ultimately, this work provides a compelling model for the development of next-generation analytical methods, not only scientifically and cost-effective, but also deeply rooted in environmental responsibility and social relevance.

## Supplementary Information

Below is the link to the electronic supplementary material.

Supplementary Material 1

## Data Availability

Data will be made available on request.
