# Supplementary material for: Sulfonated polystyrene nanospheres from waste sources for the extraction of sulfonamide antibiotics from complex matrices
Source: Mikrochim Acta. 2026 Feb 16;193(3):161. doi: 10.1007/s00604-026-07875-1 (PMC12909386; doi:10.1007/s00604-026-07875-1)
Supplement: Supplementary file 1 — Supplementary Material 1 [file 604_2026_7875_MOESM1_ESM.docx]

**Electronic Supplementary Material**

**Sulfonated polystyrene nanospheres from waste sources for the extraction of sulfonamide antibiotics from complex matrices**

Lorenzo Antonelli^a,b^, Ángela Inmaculada López-Lorente^a,*^, Alessandra Gentili^b^, Rafael Lucena^a^, Soledad Cárdenas^a,*^

^a^Affordable and Sustainable Sample Preparation (AS_2_P) research group, Departamento de Química Analítica, Instituto Químico para la Energía y el Medioambiente IQUEMA, Universidad de Córdoba, Campus de Rabanales, Edificio Marie Curie, E-14071, Córdoba, Spain.

^b^ Department of Chemistry, Sapienza University, P.le Aldo Moro 5, 00185, Rome, Italy.

*Corresponding author e-mail: [q32loloa@uco.es](mailto:q32loloa@uco.es) (A.I. López-Lorente); [qa1caarm@uco.es](mailto:qa1caarm@uco.es) (S. Cárdenas)

***Table S1*** *Physicochemical properties, retention times, and mass spectrometric transitions of selected sulfonamidesre*

| **Common name** | **Structure** | **Exact mass (u)** | **logD^a^** | **pKa_1_^b^** | **pKa_2_^c^** | **Retention time (min)** | **1^st^ transition**  **(m/z)** | **2^nd^ transition (m/z)** |
| --- | --- | --- | --- | --- | --- | --- | --- | --- |
| Sulfamerazine | 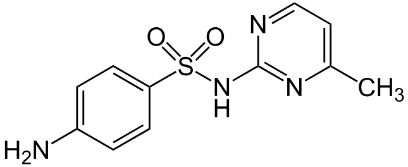 | 264.0681 | 0.50 | 2 | 6.99 | 2.6 | 265/156 | 265/92.2 |
| Sulfadiazine | 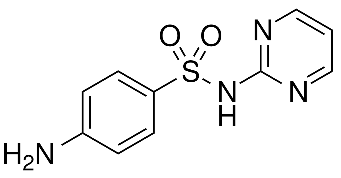 | 250.0524 | 0.37 | 2.01 | 6.99 | 2.9 | 251/92.2 | 251/65.3 |
| Sulfaguanidine | 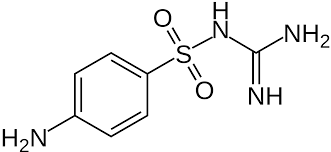 | 214.0524 | -2.95 | 7.72 | 10.53 | 5.1 | 215/92.2 | 215/65.2 |
| Sulfanilamide | 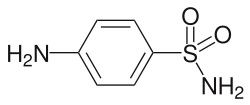 | 172.0306 | -0.27 | 2.27 | 10.99 | 5.5 | 173/56 | 173/92 |

***^a^*** *at pH 3.5*

***^b^*** *pKa_1_ is related to the dissociation of the aromatic amino group (strongest basic pKa)*

***^c^*** *pKa_2_ is related to the dissociation of the sulfonamide functional group (strongest acidic pKa)*

*Data of logD and pKa obtained from Chemicalize.*


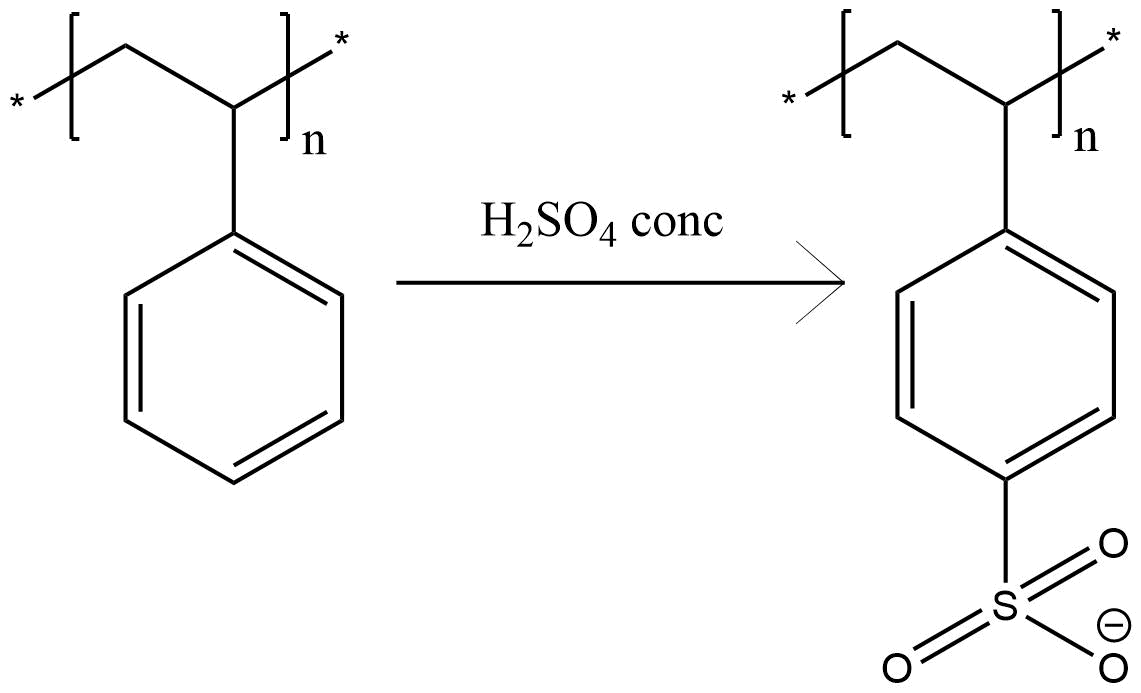


***Fig. S1*** *Sulfonation process of polystyrene*

**ATR-FTIR characterization of the materials**

The FTIR spectrum of polystyrene displays a distinctive absorption band around 3020 cm⁻¹, corresponding to =C–H stretching vibrations of the aromatic ring. The bands at 2918 cm⁻¹ and 2846 cm⁻¹ are assigned to the asymmetric and symmetric stretching modes of CH₂ groups, respectively. Furthermore, prominent peaks at 1602 cm⁻¹, 1490 cm⁻¹, and 1450 cm⁻¹ reflect the C=C stretching vibrations within the benzene ring. An absorption band near 1025 cm⁻¹ is attributed to C–O stretching, while bands in the 680–800 cm⁻¹ range are associated with the out-of-plane rocking vibrations of C–H bonds in the aromatic ring.


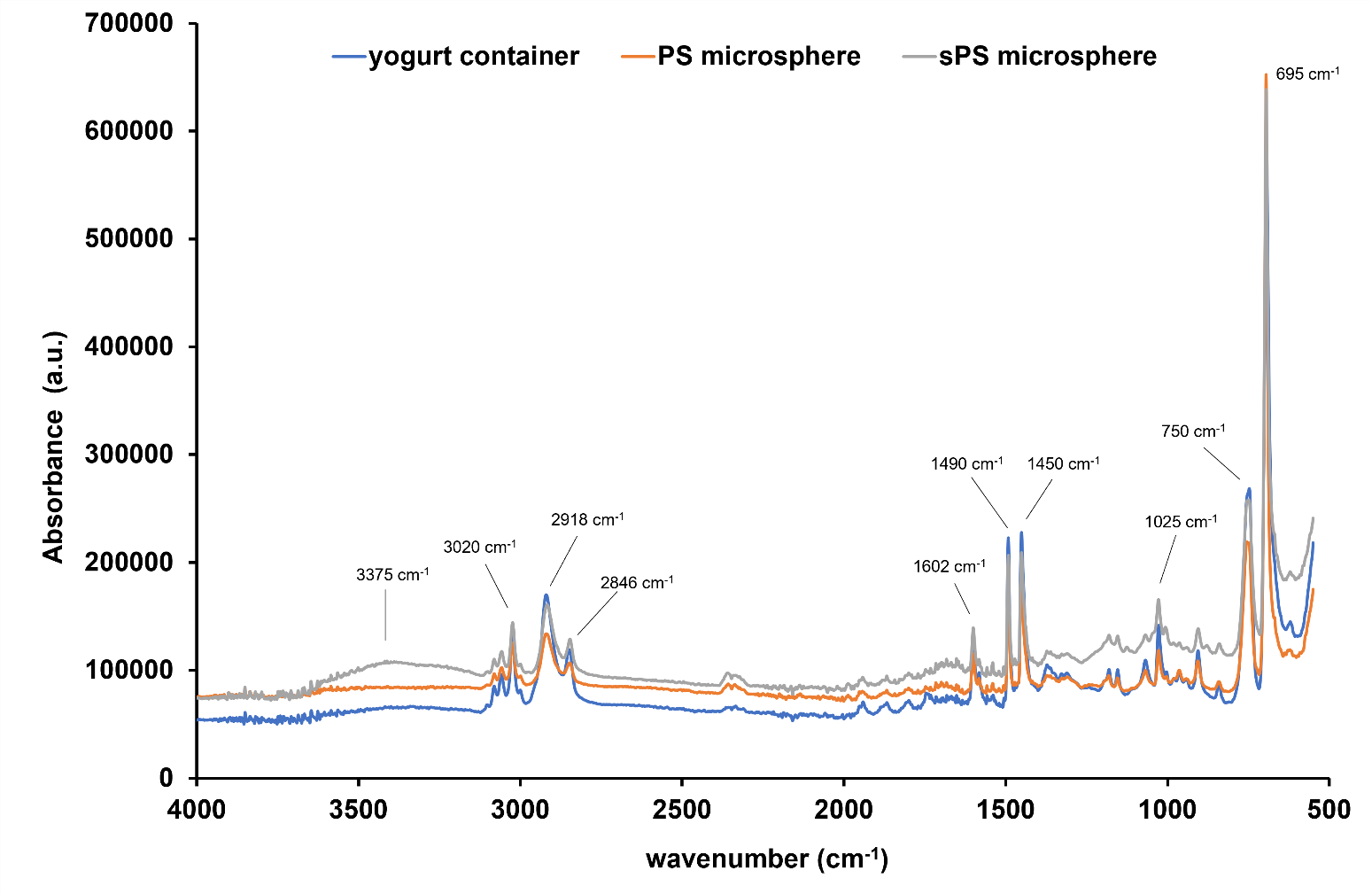


***Fig. S2*** *ATR-FTIR spectra of polystyrene waste, pristine polystyrene microbeads, and sulfonated microbeads after the sulfonation process.*


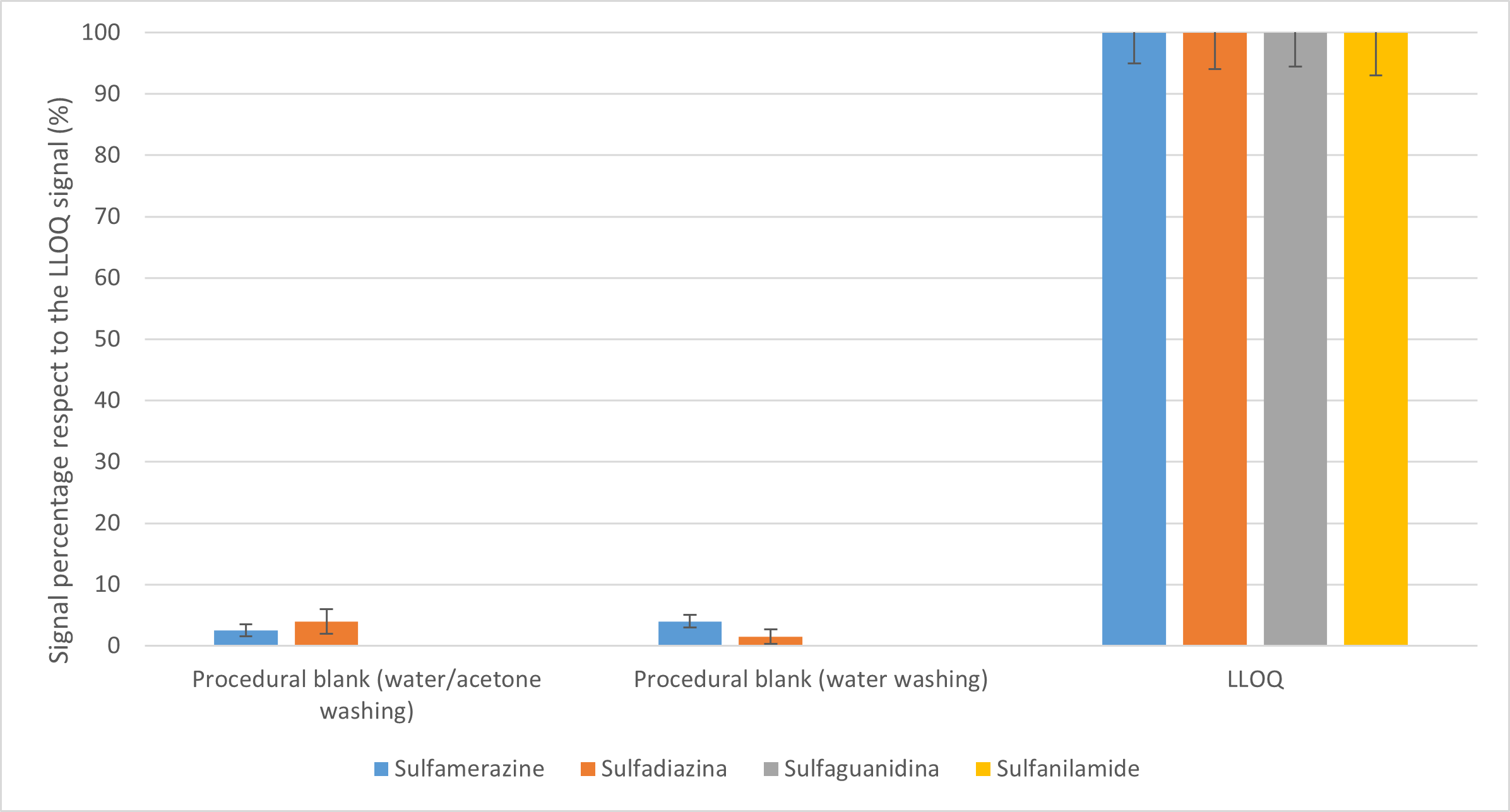


***Fig. S3*** *Effectiveness of the washing protocol procedure, resulting in a negligible memory effect of the reused materials*


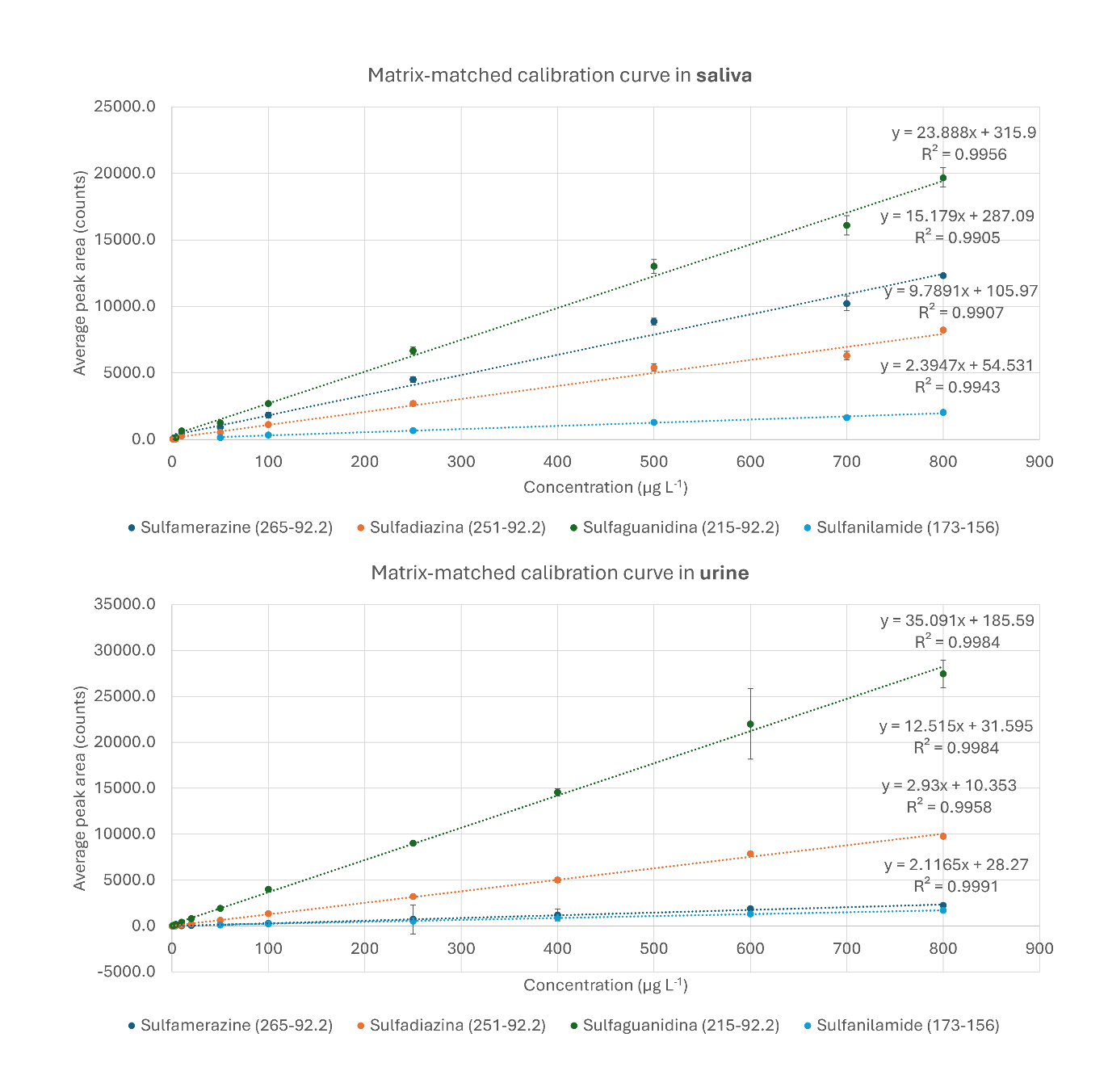


***Fig. S4*** *Matrix-matched calibration curves of the selected sulfonamides in real biological samples*

***Table S2*** *Comparison of the proposed analytical method with other articles reporting the determination of sulfadiazine (SD), sulfamerazine (SM), sulfanilamide (SA) and sulfaguanidine (SG) in biological fluids.*

| Instrumental technique | Extraction Technique | LOQ (µg L^-1^) | | | | Extraction Time (min) | Matrix | Recycling Material | Ref. |
| --- | --- | --- | --- | --- | --- | --- | --- | --- | --- |
|  |  | **SD** | **SM** | **SA** | **SG** |  |  |  |  |
| Spectrophotometric | Disk-SPME | 6.26 | 4.49 | 11.19 | - | > 20 | River water | no | [1] |
| Spectrophotometric | SPE | 130 | 100 | 60 | - | > 30 | Seawater | no | [2] |
| Smartphone-based fluorimetry | Pipette-tip SPME | - | 9.1 | - | - | n.a. | Environmental water | no | [3] |
| HPLC-DAD | SPE with 3D-printed device | 20 | 3 | - | - | 40 | Environmental water | no | [4] |
| HPLC-MS/MS | LLLME | - | - | 0.22 | - | ⁓ 60 | Wastewater | no | [5] |
| HPTLC | MISPE | 10 | - | - | 10 | ⁓ 60 | Environmental water | no | [6] |
| LC‑MS/MS | QuEChERS | 5-12  (ng g^-1^) | 5-13  (ng g^-1^) | 3-10  (ng g^-1^) | 3-10  (ng g^-1^) | ⁓ 70 | Animal tissues and products | no | [7] |
| HILIC-QTOF-MS | dSPE | 5  (ng g^-1^) | 5  (ng g^-1^) | - | - | > 3 h | Food product | no | [8] |
| HPLC-MS/MS | IS-dSPME | 4-5 | 4-5 | 50 | 2-4.8 | ⁓ 35 | Biological matrices | yes | This work |

HPLC-DAD: High-Performance Liquid Chromatography with Diode Array Detection; MS: Mass spectrometry; HPTLC: High Performance Thin-Layer Chromatography; HILIC-QTOF-MS: Hydrophilic Interaction Liquid Chromatography - Quadrupole Time-of-Flight Mass Spectrometry; SPME: solid-phase microextraction; SPE: solid-phase extraction; LLLME: liquid–liquid–liquid microextraction; MISPE: Molecularly Imprinted Polymer Solid-Phase Extraction; dSPE: dispersive solid phase extraction; IS-dSPME: in-syringe dispersive solid-phase microextraction.

**References**

[1] Peixoto, P. S., Tóth, I. V., Machado, S., Barreiros, L., Machado, A., Bordalo, A. A., Lima, J. L. F. C., Segundo, M. A. (2018). Screening of sulfonamides in waters based on miniaturized solid phase extraction and microplate spectrophotometric detection. *Anal. Methods.*, *10*(7), 690-696. https://doi.org/10.1039/C7AY02624B

[2] Ait Errayess, S., Ait Lahcen, A., Idrissi, L., Marcoaldi, C., Chiavarini, S., Amine, A. (2017). A sensitive method for the determination of Sulfonamides in seawater samples by Solid Phase Extraction and UV–Visible spectrophotometry. *Spectrochim. Acta A Mol. Biomol. Spectrosc.*, *181*, 276-285. https://doi.org/10.1016/j.saa.2017.03.061

[3] Barzallo, D., Ferrer, L., Palacio, E. (2024). Eco-friendly screening method for sulfonamides using a 3D handheld smartphone-based fluorescence detection device and graphene nanoplatelet-packed pipette tip microextraction. *J. Environ. Chem. Eng.,* 12(2), 111888. https://doi.org/10.1016/j.jece.2024.111888

[4] Barzallo, D., Palacio, E., March, J., Ferrer, L. (2023). 3D printed device coated with solid-phase extraction resin for the on-site extraction of seven sulfonamides from environmental water samples preceding HPLC-DAD analysis. *Microchem. J.*, *190*, 108609. https://doi.org/10.1016/j.microc.2023.108609

[5] Schlüsener, M. P., Bester, K. (2005). Determination of steroid hormones, hormone conjugates and macrolide antibiotics in influents and effluents of sewage treatment plants utilising high‐performance liquid chromatography/tandem mass spectrometry with electrospray and atmospheric pressure chemical ionisation. *Rapid Commun. Mass Spectrom.*, 19(22), 3269-3278. https://doi.org/10.1002/rcm.2189

[6] Pavlović, D. M., Nikšić, K., Livazović, S., Brnardić, I., Anžlovar, A. (2015). Preparation and application of sulfaguanidine-imprinted polymer on solid-phase extraction of pharmaceuticals from water. *Talanta*, *131*, 99-107. https://doi.org/10.1016/j.talanta.2014.06.065

[7] Kim, Y. R., Park, S., Kim, J. Y., Choi, J. D., Moon, G. I. (2024). Simultaneous determination of 31 Sulfonamide residues in various livestock matrices using liquid chromatography-tandem mass spectrometry. *Appl. Biol. Chem.*, *67*(1), 13. https://doi.org/10.1186/s13765-024-00864-z

[8] Petrarca, M. H., de Campos Braga, P. A., Reyes, F. G. R., Bragotto, A. P. A. (2022). Exploring miniaturized sample preparation approaches combined with LC-QToF-MS for the analysis of sulfonamide antibiotic residues in meat-and/or egg-based baby foods. *Food Chem.*, *366*, 130587. https://doi.org/10.1016/j.foodchem.2021.130587
